# Supplementary material for: Phytochemical Composition and Antioxidant Activity of Manuka Honey and Ohia Lehua Honey
Source: Nutrients. 2025 Jan 13;17(2):276. doi: 10.3390/nu17020276 (PMC11767333; doi:10.3390/nu17020276)
Supplement: Supplementary file 1 [file nutrients-17-00276-s001.zip › nutrients-3374209-supplementary.pdf]

Phytochemical Composition and Antioxidant Activity of Manuka Honey and OhiaLehua Honey

Iulia Ioana Morar[1], Raluca Maria Pop[2*], Erik Peitzner[3], Floricuța Ranga[4], Meda Sandra Orăsan[1], Andra Diana Cecan[1], Elisabeta Ioana Chera[1], Teodora Irina Bonci[1], Lia Oxana Usatiuc[1], Mădălina Țicolea[1], Anca Elena But[1], Florinela Adriana Cătoi[1], Alina Elena Pârvu[1] and Mircea Constantin Dinu Ghergie[5]

[1]Pathophysiology, Department of Morpho-Functional Sciences, Faculty of Medicine, University of Medicine and Pharmacy "Iuliu Hațieganu" Cluj-Napoca, Romania, 400012 Cluj-Napoca, Romania; iulia.morar@umfcluj.ro; orasan.meda@umfcluj.ro; andra.cecan@umfcluj.ro; chera.elisabeta@umfcluj.ro; adam.teodora@umfcluj.ro; lia.usatiuc@umfcluj.ro; madalina.ticolea@umfcluj.ro; anca.but@umfcluj.ro; adriana.catoi@umfcluj.ro; parvualinaelena@umfcluj.ro

[2]Pharmacology, Toxicology and Clinical Pharmacology, Department of Morpho-Functional Sciences, Faculty of Medicine, "Iuliu Hațieganu" University of Medicine and Pharmacy, Cluj-Napoca, Romania, 400012 Cluj-Napoca, Romania; raluca.pop@umfcluj.ro

[3]Faculty of Medicine, University of Medicine and Pharmacy "Iuliu Hațieganu," Cluj-Napoca, Romania, 400012 Cluj-Napoca, Romania;peitzner.erik@proton.me

[4]Food Science and Technology, Department of Food Science, University of Agricultural Science and Vet-erinary Medicine Cluj-Napoca, CaleaMănăștur, No 3-5, 400372, Cluj-Napoca România; florica.ranga@usamv-cluj.ro

[5]Orthodontics, Department of Conservative Odontology, Faculty of Dental Medicine, "Iuliu Hațieganu" University of Medicine and Pharmacy, Cluj-Napoca, Romania, 400012 Cluj-Napoca, Romania; ghergie.mircea@umfcluj.ro

*Correspondence: raluca.pop@umfcluj.ro

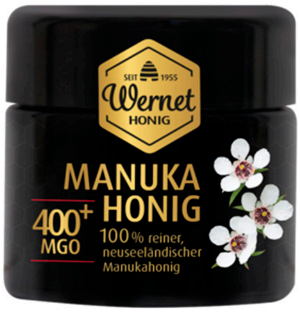

Figure S1. Manuka honey

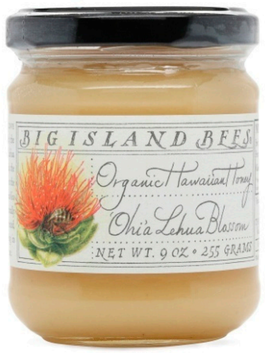

Figure S2. OhiaLehua honey

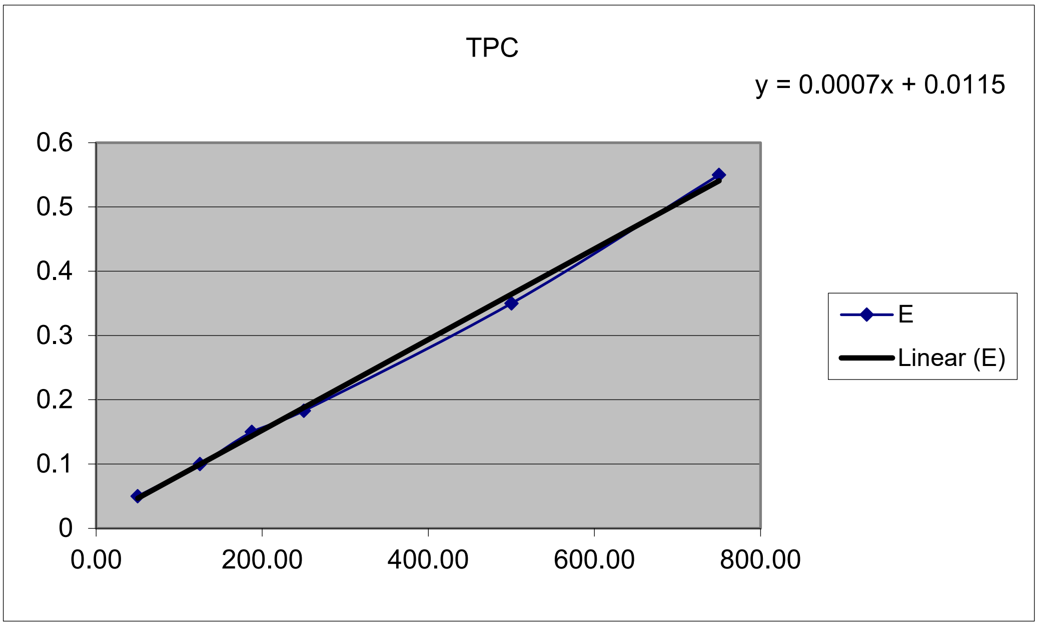

Figure S3. Gallic acid calibration curve (mg GAE/g)

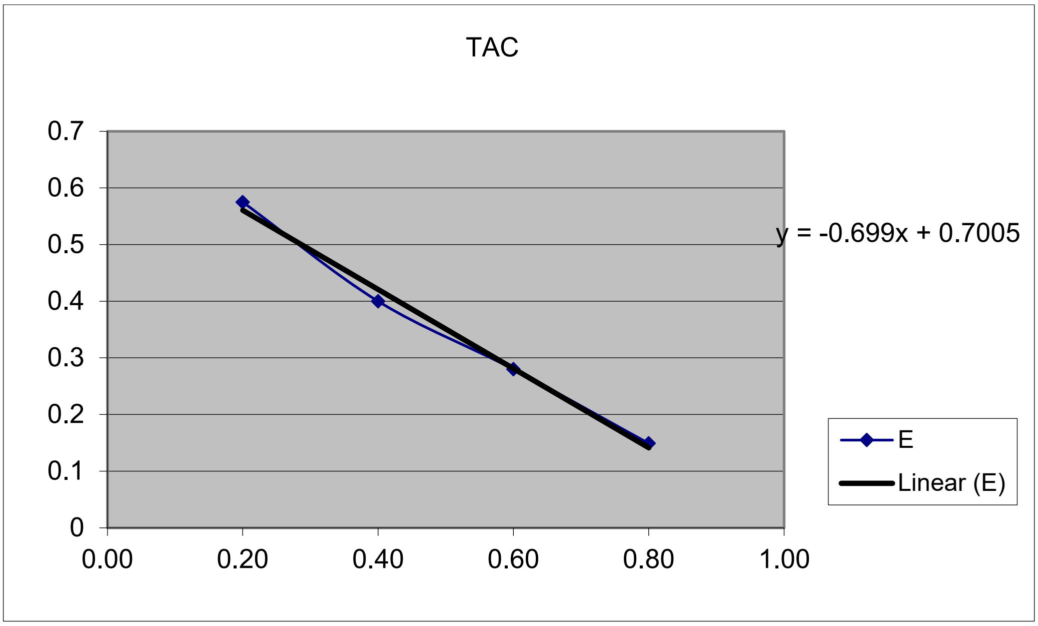

Figure S4. Trolox calibration curve (mmol TE/L)

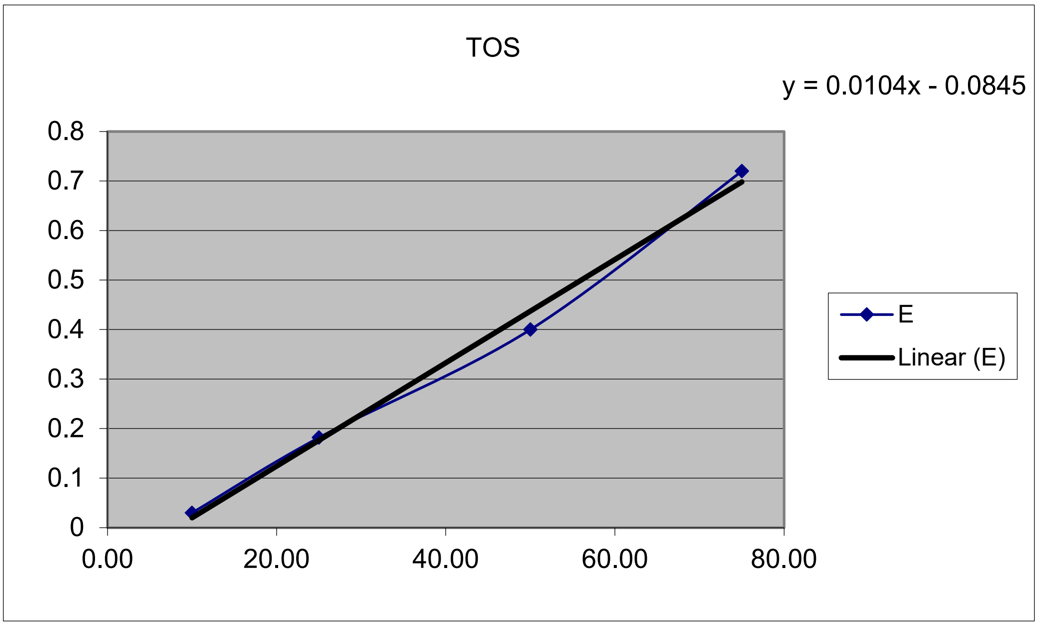

Figure S5. H_2O_2 calibration curve (μmol H_2O_2/L)

Table S1. Pearson correlations analysis of OhiaLehuaHoney 100

	TAC	*TOS*	*NO*	*MDA*	*SH*	*OSI*	*AOPP*
TAC	1						
TOS	-0.62322	1					
NO	0.086455	-0.05162	1				

MDA	-0.06583	-0.30801	0.51107	1			
SH	-0.24747	0.841349	0.3857	-0.13322	1		
OSI	-0.63604	0.99986	-0.0546	-0.30186	0.834738	1	
AOPP	-0.26742	0.382807	-0.08492	0.032174	0.75621	0.479403	1

Total antioxidant capacity (TAC), total oxidant status (TOS), nitric oxide (NO), malondialdehyde (MDA), total thiols (SH), oxidative stress index (OSI) and advanced oxidation protein products (AOPP).

Table S2. Pearson correlations analysis of OhiaLehuaHoney 50

	TAC	*TOS*	*NO*	*MDA*	*SH*	*OSI*	*AOPP*
TAC	1						
TOS	0.696594	1					
NO	0.108348	-0.50476	1				
MDA	0.228042	-0.5771	0.986162	1			
SH	0.374116	0.990736	-0.88139	-0.8176	1		
OSI	0.478099	0.999776	-0.82133	-0.7461	0.99339	1	
AOPP	0.623376	0.486809	-0.67508	-0.6714	0.958356	0.384802	1

Total antioxidant capacity (TAC), total oxidant status (TOS), nitric oxide (NO), malondialdehyde (MDA), total thiols (SH), oxidative stress index (OSI) and advanced oxidation protein products (AOPP).

Table S3. Pearson correlations analysis of OhiaLehuaHoney 25

	TAC	*TOS*	*NO*	*MDA*	*SH*	*OSI*	*AOPP*
TAC	1						
TOS	0.697648	1					
NO	0.389222	0.312692	1				
MDA	0.007004	0.713364	0.75428	1			
SH	0.445894	0.932398	0.387759	0.604402	1		
OSI	0.69469	0.999991	0.312184	0.415421	0.933641	1	
AOPP	0.752345	0.311902	0.57672	0.593337	0.819406	0.411406	1

Total antioxidant capacity (TAC), total oxidant status (TOS), nitric oxide (NO), malondialdehyde (MDA), total thiols (SH), oxidative stress index (OSI) and advanced oxidation protein products (AOPP).

Table S4. Pearson correlations analysis of Manuka Honey 100

	TAC	*TOS*	*NO*	*MDA*	*SH*	*OSI*	*AOPP*
TAC	1						
TOS	0.682554	1					
NO	-0.6035	-0.66828	1				
MDA	-0.67018	-0.26316	0.116773	1			
SH	-0.18999	0.706877	-0.05624	0.756029	1		
OSI	0.679357	0.99999	-0.46643	-0.26032	0.409602	1	
AOPP	0.491541	0.766071	0.137789	-0.50585	0.116169	0.766376	1

Total antioxidant capacity (TAC), total oxidant status (TOS), nitric oxide (NO), malondialdehyde (MDA), total thiols (SH), oxidative stress index (OSI) and advanced oxidation protein products (AOPP).

Table S5. Pearson correlations analysis of Manuka Honey 50

	TAC	*TOS*	*NO*	*MDA*	*SH*	*OSI*	*AOPP*
TAC	1						
TOS	-0.13192	1					
NO	-0.01112	-0.66987	1				
MDA	0.82826	-0.17861	-0.37084	1			
SH	0.598748	-0.58099	0.792808	0.186362	1		
OSI	-0.14051	0.999962	-0.66859	-0.18593	-0.5852	1	
AOPP	0.970044	-0.21347	0.71944	0.935742	0.483574	-0.2218	1

Total antioxidant capacity (TAC), total oxidant status (TOS), nitric oxide (NO), malondialdehyde (MDA), total thiols (SH), oxidative stress index (OSI) and advanced oxidation protein products (AOPP).

Table S6. Pearson correlations analysis of Manuka Honey 25

	TAC	*TOS*	*NO*	*MDA*	*SH*	*OSI*	*AOPP*
TAC	1						
TOS	0.487486	1					
NO	0.399118	0.664484	1				
MDA	0.853051	0.419753	-0.04448	1			
SH	0.404725	0.593463	-0.18362	0.761559	1		
OSI	0.483055	0.999987	0.664047	0.415977	0.592776	1	

AOPP	-0.54166	-0.99596	-0.62075	-0.49856	-0.64938	-0.9956	1
